# Antiviral efficacy and safety of abacavir-containing combination antiretroviral therapy as first-line treatment of HIV-infected children and adolescents: a systematic review protocol

**DOI:** 10.1186/2046-4053-3-87

**Published:** 2014-08-12

**Authors:** Olatunji O Adetokunboh, Anel Schoonees, Charles S Wiysonge

**Affiliations:** 1 Centre for Evidence-based Health Care, Faculty of Medicine and Health Sciences, Stellenbosch University, Cape Town 7505, South Africa; 2 Division of Community Health, Faculty of Medicine and Health Sciences, Stellenbosch University, Cape Town 7505, South Africa

**Keywords:** Abacavir, Children, Adolescents, Antiretroviral therapy, HIV, Efficacy, Safety

## Abstract

**Background:**

Abacavir is one of the recommended nucleoside reverse transcriptase inhibitors (NRTIs) for the treatment of HIV infections among children and adolescents. However, there are concerns that the antiviral efficacy of abacavir might be low when compared to other NRTIs especially among children. There are also concerns that abacavir use may lead to serious adverse events such as hypersensitivity reactions and has potential predisposition to developing cardiovascular diseases.

**Methods:**

We plan to do a systematic review to evaluate the antiviral efficacy and safety of abacavir-containing combination antiretroviral therapy as first-line treatment of HIV-infected children aged between 3 months and 18 years, compared with other NRTIs. We will search Scopus, Cochrane Central Register of Controlled Trials, MEDLINE, and Web of Science databases for eligible studies regardless of language or publication status. We will check the reference lists of included studies, search relevant conference proceedings, email the authors of included studies and also look for unpublished and ongoing trials in prospective clinical trial registries. Two authors will independently screen search outputs, select studies, extract data and assess the risk of bias in included studies. All disagreements will be resolved by discussion and consensus. Where data allow, we will conduct meta-analysis for similar types of participants, study designs, interventions, and outcome measures. If the results are statistically homogeneous, we will use the fixed-effect model; otherwise, we will use the random-effects model and explore the reasons for heterogeneity using subgroup analyses. Heterogeneity will be assessed with the Chi-squared test and quantified with the I-squared statistic.

**Discussion:**

The findings will be useful to policy makers and programme managers to inform treatment and management of HIV in children and adolescents and to point out research gaps for future research.

**Trial registration:**

This review is registered with PROSPERO, registration number CRD42014009157.

## Background

The acquired immunodeficiency syndrome (AIDS) remains a major global concern with estimated 3.3 million under 15 years of age children and adolescents currently living with the human immunodeficiency virus (HIV) and about 2 million needing antiretroviral therapy [1]. In order to effectively manage HIV infection and AIDS, it is recommended that antiretroviral treatment regimens should consist of a three-drug combination consisting of two nucleoside reverse transcriptase inhibitors (NRTIs) with either one protease inhibitor (PI) or a non-nucleoside reverse transcriptase inhibitor (NNRTI) depending on the age of the patient and other co-morbidities [2]. In the treatment of children infected by HIV, abacavir is one of the recommended NRTIs in children younger than 10 years of age [2]. Abacavir, formerly known as 1592U89, is a carbocyclic 2′-deoxyguanosine nucleoside analogue with its main activity being against HIV type 1 (HIV-1). It is phosphorylated to its active metabolite, carbovir triphosphate, which inhibits the HIV-1 reverse transcriptase competitively and terminates deoxyribonucleic acid (DNA) synthesis. This prevents HIV from replicating, thereby lowering the amount of HIV in the body system [3].

The two NRTIs in a three-drug antiretroviral regimen are referred to as the NRTI backbones of the regimen. As shown in Table 1, the World Health Organization (WHO) recommends abacavir and either lamivudine or zidovudine as the NRTI backbones for children younger than 3 years of age. There is a strong recommendation for the use of these two NRTI backbones as fixed-dose combinations in this paediatric age group; however, this recommendation was done with a low certainty of evidence [2]. In 3- to 10-year-old children and adolescents weighing less than 35 kg, abacavir-lamivudine is the NRTI backbone commonly used. Again, this regimen has strong recommendation for use but low certainty of evidence [2].

**Table 1:** **Summary of WHO recommended first-line antiretroviral regimens for children and adolescents**[2]

	**Children 3 years to less than 10 years and adolescents <35 kg**	**Adolescents (10 to 19 years)** ≥**35 kg**
Preferred	ABC^a^ + 3TC + EFV	TDF + 3TC (or FTC) + EFV^a^
Alternatives	ABC + 3TC + NVP	
	AZT + 3TC + EFV	AZT + 3TC + EFV
	AZT + 3TC + NVP	AZT + 3TC + NVP
	TDF + 3TC (or FTC) + EFV	TDF + 3TC (or FTC) + NVP
	TDF + 3TC (or FTC) + NVP	
Special circumstances^c^	d4T^b^ + 3TC + EFV	ABC + 3TC + EFV
	d4T^b^ + 3TC + NVP	ABC + 3TC + NVP

^a^These recommendations apply to children and adolescents who are initiating first-line antiretroviral therapy; ^b^d4T use should be restricted to situations in which toxicity to AZT is suspected or confirmed and access to ABC or TDF is lacking.

^c^Special circumstances may include situations where preferred or alternative regimens may not be available or suitable because of significant toxicities, anticipated drug-drug interactions, drug procurement and supply management issues, or for other reasons.

*3TC* lamivudine, *ABC* abacavir, *AZT* zidovudine, *d4T* stavudine, *EFV* efavirenz, *FTC* emtricitabine, *LPV/r* lopinavir/ritonavir, *NVP* nevirapine, *TDF* tenofovir, *WHO* World Health Organization.

The WHO guidelines suggest that stavudine, a NRTI, be replaced by abacavir because of toxicity concerns. However, abacavir has adverse effect concerns of its own [4]. Abacavir is associated with a systemic illness known as *abacavir hypersensitivity reaction* that can result in death if the drug is not discontinued in affected patients. This hypersensitivity may present with fever, maculopapular rash and other constitutional symptoms such as fatigue, malaise and myalgia. Gastrointestinal adverse effects such as vomiting, diarrhoea and abdominal pain may also occur. Occasionally, there are also some prominent respiratory symptoms, such as tachypnea and cough [5,6]. Hypersensitivity reactions due to abacavir have been reported in both paediatric and adult populations with the incidence in randomised controlled trials ranging from 0% to 14% [6]. HIV-infected individuals of African descent seems to have reduced risk of abacavir hypersensitivity [7], and the wide variation in reported adverse event incidence with abacavir use makes it necessary to do a systematic review, especially in children. In addition, some cohort studies in South Africa have shown poor virological responses to abacavir-based regimens when compared to stavudine in children. These studies queried the clinical effectiveness of abacavir when compared to the other NRTIs as well as the justification for making it a first-line drug in the treatment of HIV in children [8,9]. A further investigation on the drug is thus needed.

Some research studies suggested that abacavir increases the risk of cardiovascular events, especially myocardial infarction [10,11]. However, meta-analyses of randomised controlled trials in adults have not supported the postulation that abacavir-containing antiretroviral regimens carry a greater risk of cardiovascular events relative to abacavir-sparing regimens [12,13]. Similarly, various studies evaluating changes in inflammatory and coagulopathic biomarkers upon commencement of abacavir-containing regimens have produced conflicting findings [14,15]. These randomised controlled trials were carried out mainly on adults due to the belief that children have lower incidence of some of these important adverse effects of abacavir [16]. A meta-analysis of HIV-infected adults switching to abacavir-containing regimens shows rather weak evidence of lower incidence of adverse events, with higher incidence of virological failure in the NRTI groups when compared to the controls [17].

Despite concerns that the confidence in the currently available evidence on the antiviral efficacy of abacavir might be low, coupled with serious adverse events such as hypersensitivity reactions and a potential predisposition to developing cardiovascular diseases, the WHO has recommended abacavir as one of the preferred NRTI backbones in the paediatric population [4]. However, we are not aware of any systematic review of the safety of abacavir-containing regimens in HIV-infected children.

### Objective

The primary objective is to assess the antiviral efficacy of abacavir-containing combination antiretroviral regimens in comparison with combination antiretroviral regimens containing other NRTIs as first-line therapy for HIV-infected children and adolescents.

The secondary objective is to assess the safety of abacavir-containing combination antiretroviral regimens in HIV-infected children and adolescents.

## Methods

This review protocol has been published in the PROSPERO International Prospective Register of systematic reviews (http://www.crd.york.ac.uk/PROSPERO), registration number CRD42014009157 [18]. The PROSPERO database provides a comprehensive listing of systematic reviews registered at the start to help prevent unintended duplication and allows comparison of published review methods with what was intended in the protocol.

### Criteria for considering studies for this review

#### ***Types of studies***

Experimental (randomised controlled trials and non-randomised controlled trials) and prospective observational studies with control groups will be eligible for inclusion in this systematic review. Non-randomised controlled trials refer to studies that allocated participants to interventions and controls using alternation between groups, by the use of birth dates or weekdays or by other non-random methods.

#### ***Types of participants***

HIV-infected individuals between 3 months and 18 years of age.

#### ***Types of interventions***

Experimental group: abacavir-containing combination antiretroviral regimens as first-line therapy.

Control group: combination antiretroviral regimens containing zidovudine or tenofovir or stavudine in the NRTI backbone as first-line therapy.

#### ***Types of outcome measures***

Primary outcomes:

1. Virological suppression

2. Virological failure

Secondary outcomes:

1. Adverse events requiring treatment interruption or switching

2. CD4 cell count

3. Hypersensitivity reaction (fever, nausea, respiratory discomfort, rash and diarrhoea)

4. Death (all cause)

5. Myocardial infarction and other cardiovascular events

### Search methods for identification of studies

With the support of a librarian at the University of Stellenbosch's Faculty of Medicine and Health Sciences, we will do an exhaustive search to identify all the relevant studies regardless of language or publication status.

#### ***Databases***

We will search the following electronic databases: MEDLINE via PubMed, Cochrane Central Register of Controlled Trials (CENTRAL), Scopus, and ISI Web of Science (Science Citation Index). Both text words and medical subject heading (MeSH) terms, for example abacavir, antiretroviral, HIV, acquired immunodeficiency syndrome, child, paediatric, adolescent and randomised controlled trial will be used in the search strings. We will use these terms in different combinations with the adaptation of the literature search strategy to suit each database (see Additional file 1 for the proposed search strategy adapted from Shey *et al*. [19]).

#### ***Conference proceedings***

We will search the proceedings of the following conferences for potentially eligible studies: The European AIDS Clinical Society (EACS) conferences, International AIDS conferences, Conference on Retroviruses and Opportunistic Infections (CROI) and International AIDS Society conference on HIV Pathogenesis and Treatment (IAS).

#### ***Other resources***

We will search for unpublished and ongoing studies in prospective clinical trial registries such as ClinicalTrials.gov and WHO International Clinical Trials Registry Platform. In addition, we will contact authors of studies included in this review to find out if they know of any other relevant studies in the field. Lastly, we will search the reference lists of included studies and relevant systematic reviews.

### Data collection and analysis

The data collection and analysis for this review will be based on standard Cochrane Collaboration methods [20].

#### ***Selection of studies***

Two members of the research team will independently screen the titles and abstracts identified by the literature searches and identify all potentially eligible studies. We will then obtain the full text articles for these and review them independently. We will resolve all disagreements by discussion and consensus within the author team. Reasons for excluding studies previously identified as potentially eligible will be recorded in the table of excluded studies.

#### ***Data extraction and management***

Two members of the team will independently extract data using a pre-piloted data extraction form. The following data will be extracted:

a. Details of the study (setting, study design, date and time period)

b. Details of participants (age, gender, co-morbidities, relevant baseline characteristics)

c. Details of the treatment and control interventions, including dose and exact regimens

d. Number of participants randomised per group and number of withdrawals per group with reasons

e. Numerical results for each of our pre-specified outcomes

f. Conflicts of interest declarations and funding sources

In case of missing or unclear information in any of the included studies, we will try to get further information or clarity from the trial authors via email. The two authors will compare extracted data for each study and resolve any differences by discussion and consensus, with the third author arbitrating if necessary. One author will enter the final data into the latest version of the Cochrane Collaboration Review Manager, currently version 5.2 (RevMan 5.2) [21], and the second author will cross-check the data for data entry errors and rectify where needed.

#### ***Assessment of risk of bias in included studies***

Two authors will independently assess the risk of bias in each included experimental study using the domain-based tool as described in the Cochrane Handbook for Systematic Reviews of Interventions 5.1.0 [20]. The following domains will be assessed: random sequence generation, allocation concealment, blinding of participants and personnel, blinding of outcome assessors, incomplete outcome data, selective reporting and other biases (baseline imbalances, industry involvement and conflicts of interest). Judgements will be made as low risk of bias, unclear risk of bias or high risk of bias according to the criteria in the Cochrane Handbook [20]. Two authors will compare the assessments and resolve differences by discussion and consensus. In case of failure to resolve the difference, a third author will be called upon to arbitrate. RevMan 5.2 will be used to display the results of the risk of bias assessment per study and overall per domain [20].

Regarding included prospective observational studies, the two authors will also independently evaluate the methodological quality by using the Newcastle-Ottawa Quality Assessment Scale [22]. Emphasis will be placed on the method of selection of the sample, the type, length and completeness of follow-up and objectivity of outcome assessment.

##### 

**Measures of treatment effect** Dichotomous data will be expressed as risk ratios (RR) and continuous data as mean differences (MD).

##### 

**Dealing with missing data** Where information on results is unclear or missing, we will get in touch with trial authors via email. Where appropriate, data will be analysed with both the intention-to-treat (ITT) and available case principles.

##### 

**Assessment of heterogeneity** Data will only be pooled from studies judged to be largely homogenous in trial participants, interventions and various outcomes. We will visually inspect the forest plots to check for overlapping of intervention effects across the included studies. Following the visual inspection, we will conduct the Chi-squared test of homogeneity to formally test for heterogeneity of study effects, defining significance at *α* = 10%. In addition, we will use the I-squared statistic to quantify heterogeneity of effects among the trials, where an I-square value above 50% is seen as substantial heterogeneity [20].

##### 

**Quality of evidence** The quality of the evidence (also referred to as certainty of the evidence, confidence in the estimate or strength of the evidence) is an assessment suggesting the confidence we can place in the findings. We will assess the quality of our review's evidence by using the Grading of Recommendations Assessment, Development and Evaluation (GRADE) tool and incorporate the GRADE profiles in ‘summary of findings’ tables. The GRADE approach results in judging the evidence per outcome as high, moderate, low or very low [23,24]. Reasons for downgrading or upgrading evidence, for randomised controlled trails and observational studies respectively, will be provided.

##### 

**Assessment of reporting biases** In the case of at least ten studies included in a meta-analysis, we will assess the likelihood of publication bias for the outcome(s) using funnel plot techniques [20].

##### 

**Data synthesis** Data analysis will be conducted using the latest RevMan software (currently version 5.2) [21]. Where included studies are found to be clinically and statistically homogeneous, they will be pooled in a meta-analysis. Effect sizes will be reported alongside 95% confidence intervals.

Meta-analysis will be done using the fixed-effect model when the Chi-squared test yields *P* > 0.1 or when the I-squared value is below 50%; otherwise, the random-effects model will be used. When the I-squared value is greater than 75%, indicating considerable heterogeneity [20], results of individual studies will not be pooled. If we are unable to conduct meta-analyses, a narrative synthesis of the results per outcome will be done or results will be provided in a table.

##### 

**Subgroup analysis and investigation of heterogeneity** We propose to conduct subgroup analyses, with subgroups defined by type of antiretroviral agents in the intervention or control groups, age group (infants, children and adolescents) and study setting (low-income, middle-income and high-income countries). These analyses will only be performed for the primary outcomes.

##### 

**Sensitivity analysis** Sensitivity analyses will be conducted to assess the effects of risk of bias and different statistical methods employed in the meta-analyses for the primary outcomes. Regarding risk of bias, our focus will be on allocation concealment (with and without studies with high or unclear risk), blinded outcome assessment (with and without studies with high or unclear risk) and losses to follow-up (with and without studies that had ≥30% attrition or differential attrition). Regarding the potential effects of different statistical methods, we will compare results of ITT and available case analysis.

##### 

**Presenting and reporting of results** We will display the study selection process by means of a flow diagram and provide reasons for exclusion of studies. We will follow the PRISMA guidelines for reporting of systematic reviews [25]. The search strings will be published as supplementary documents. We will also display the risk of bias tables, forest plots and summary of findings table(s) as appropriate.

## Discussion

To the best of our knowledge, there are no published systematic reviews that have specifically assessed the antiviral efficacy and safety of abacavir-containing combination antiretroviral regimens in the paediatric population. There are however systematic reviews on abacavir-containing combination antiretroviral regimens in adults [12,13,16,19]. The study team plans to address this gap with the proposed systematic review outlined in this protocol. The summary of the available scientific evidence would shed light on the antiviral performance of abacavir as first-line treatment of HIV-infected children and adolescents.

This study may also inform decision makers about abacavir's risk of adverse events, including cardiovascular events. Understanding the virological performance of abacavir in HIV-infected children in comparison to the less preferred antiretroviral agents is crucial in order to prevent suboptimal long-term outcomes. We also hope to identify gaps in the research that may form the basis for future studies of various aspects of abacavir in the paediatric population. The findings of this review are to be used by decision makers to inform treatment policy and paediatric HIV management practices.

## Abbreviations

ABC: abacavir; AIDS: acquired immunodeficiency syndrome; AZT: zidovudine; CENTRAL: Cochrane Central Register of Controlled Trials; CROI: Conference on Retroviruses and Opportunistic Infections; DNA: deoxyribonucleic acid; EACS: European AIDS Clinical Society; EFV: efavirenz; FTC: emtricitabine; GRADE: Grading of Recommendations Assessment, Development and Evaluation; HIV: human immunodeficiency virus; IAS: International AIDS Society; MeSH: medical subject heading; NVP: nevirapine; RevMan: Review Manager; RCT: randomised controlled trial; d4T: stavudine; TDF: tenofovir; WHO: World Health Organization.

## Competing interests

The authors declare that they have no competing interests.

## Authors’ contributions

OOA developed the study question. All the authors contributed to the design of the protocol. All authors read and approved the final manuscript.

## Supplementary Material

Additional file 1: Medline search strategy using PubMed. Click here for file

## References

[B1] WHO Global update on HIV treatment 2013: results, impact and opportunities: WHO report in partnership with UNICEF and UNAIDS 2013 Geneva, Switzerland: World Health Organization http://www.unaids.org/en/media/unaids/contentassets/documents/epidemiology/2013/gr2013/UNAIDS_Global_Report_2013_en.pdf

[B2] UNAIDS Global Report: UNAIDS Report on The Global AIDS Epidemic 2013 2013 Geneva, Switzerland: Joint United Nations Programme on HIV/AIDS http://www.who.int/hiv/data/global_treatment_report_presentation_2013.pdf 10.1016/S0968-8080(09)34466-3 19962651

[B3] Daluge SM Good SS Faletto MB Miller WH St Clair MH Boone LR Tisdale M Parry NR Reardon JE Dornsife RE Averett DR Krenitsky TA 1592U89, a novel carbocyclic nucleoside analog with potent, selective anti-human immunodeficiency virus activity Antimicrob Agents Chemother 1997 41 1082 1093 9145874 10.1128/aac.41.5.1082 PMC163855

[B4] WHO Antiretroviral Therapy for HIV Infection in Infants and Children: Towards Universal Access. Recommendations for a Public Health Approach 2006 Geneva, Switzerland: World Health Organisation http://www.who.int/hiv/pub/guidelines/art/en/23741772

[B5] Hewitt RG Abacavir hypersensitivity reaction Clin Infect Dis 2002 34 1137 1142 11915004 10.1086/339751

[B6] Clay PG The abacavir hypersensitivity reaction: a review Clin Ther 2002 24 1502 1514 12462283 10.1016/s0149-2918(02)80057-1

[B7] Nahirya-Ntege P Musiime V Naidoo B Bakeera-Kitaka S Nathoo K Munderi P Mugyenyi P Kekitiinwa A Bwakura-Dangarembizi MF Crawley J ARROW Trial Team Low incidence of abacavir hypersensitivity reaction among African children initiating antiretroviral therapy Pediatr Infect Dis J 2011 30 535 537 21164384 10.1097/INF.0b013e3182076864

[B8] Technau KG Lazarus E Kuhn L Abrams EJ Sorour G Strehlau R Reubenson G Davies MA Coovadia A Poor early virologic performance and durability of abacavir-based first-line regimens for HIV-infected children Pediatr Infect Dis J 2013 32 851 855 23860481 10.1097/INF.0b013e31828c3738 PMC3717192

[B9] Technau KG Schomaker M Kuhn L Moultrie H Coovadia A Eley B Rabie H Wood R Cox V Vizcaya LS Muchiri E Davies MA for the IeDEA Southern Africa Paediatric Collaboration Virologic response in children treated with abacavir compared with stavudine-based antiretroviral treatment – a South African multi-cohort analysis Pediatr Infect Dis J 2014 33 617 622 24378944 10.1097/INF.0000000000000222 PMC4024348

[B10] Choi AI Vittinghoff E Deeks SG Weekley CC Li Y Shlipak MG Cardiovascular risks associated with abacavir and tenofovir exposure in HIV-infected persons AIDS 2011 25 1289 1298 21516027 10.1097/QAD.0b013e328347fa16 PMC3910369

[B11] Sabin CA Worm SW Weber R Reiss P El-Sadr W Dabis F De Wit S Law M D'Arminio Monforte A Friis-Moller N Kirk O Pradier C Weller I Phillips AN Lundgren JD D:A:D Study Group Use of nucleoside reverse transcriptase inhibitors and risk of myocardial infarction in HIV-infected patients enrolled in the D:A:D study: a multi-cohort collaboration Lancet 2008 371 1417 1426 18387667 10.1016/S0140-6736(08)60423-7 PMC2688660

[B12] Cruciani M Zanichelli V Serpelloni G Bosco O Malena M Mazzi R Mengoli C Parisi SG Moyle G Abacavir use and cardiovascular disease events: a meta-analysis of published and unpublished data AIDS 2011 25 1993 2004 21716077 10.1097/QAD.0b013e328349c6ee

[B13] Ding X Andraca-Carrera E Cooper C Miele P Kornegay C Soukup M Marcus KA No association of abacavir use with myocardial infarction: findings of an FDA meta-analysis J Acquir Immune Defic Syndr 2012 61 441 447 22932321 10.1097/QAI.0b013e31826f993c

[B14] Strategies for Management of Anti-Retroviral Therapy/INSIGHT, DAD Study Groups Use of nucleoside reverse transcriptase inhibitors and risk of myocardial infarction in HIV-infected patients AIDS 2008 22 F17–F24 18753925 10.1097/QAD.0b013e32830fe35e PMC2644883

[B15] Martin A Amin J Cooper DA Carr A Kelleher AD Bloch M Baker D Woolley I Emery S STEAL study group Abacavir does not affect circulating levels of inflammatory or coagulopathic biomarkers in suppressed HIV: a randomized clinical trial AIDS 2010 24 2657 20827168 10.1097/QAD.0b013e32833f147f

[B16] Cruciani M Martí-Carvajal AJ Mengoli C Serpelloni G Bovo C Moyle G Abacavir versus other nucleoside reverse transcriptase inhibitor (NRTI) backbone therapies for treatment of HIV infection Cochrane Database Syst Rev 2011 11 CD009390

[B17] Cruciani M Mengoli C Serpelloni G Parisi SG Malena M Bosco O Abacavir-based triple nucleoside regimens for maintenance therapy in patients with HIV Cochrane Database Syst Rev 2013 6 CD008270 23740608 10.1002/14651858.CD008270.pub2 PMC11330272

[B18] Adetokunboh O Schoonees A Wiysonge CS Antiviral efficacy and safety of abacavir-containing combination antiretroviral therapy as first-line treatment of HIV infected children and adolescents: a systematic review protocol PROSPERO 2014 CRD42014009157 http://www.crd.york.ac.uk/PROSPERO/display_record.asp?ID=CRD42014009157 10.1186/2046-4053-3-87 PMC4137106 25115243

[B19] Shey MS Kongnyuy EJ Alobwede SM Wiysonge CS Co-formulated abacavir-lamivudine-zidovudine for initial treatment of HIV infection and AIDS Cochrane Database Syst Rev 2013 3 CD005481 23543540 10.1002/14651858.CD005481.pub3 PMC7026626

[B20] Higgins JPT Green S Cochrane Handbook for Systematic Reviews of Interventions Version 5.1.0 [updated March 2011] 2011 The Cochrane Collaboration http://www.cochrane-handbook.org

[B21] Review Manager (RevMan) [Computer program] The Nordic Cochrane Centre 2012 Copenhagen: The Cochrane Collaboration [Version 5.2]

[B22] The Newcastle-Ottawa Scale (NOS) for assessing the quality of nonrandomised studies in meta-analyses http://www.ohri.ca/programs/clinical_epidemiology/oxford.asp

[B23] Balshem H Helfand M Schunemann HJ Oxman AD Kunz R Brozek J Vist GE Falck-Ytter Y Meerpohl J Norris S Guyatt GH GRADE guidelines: 3. Rating the quality of evidence J Clin Epidemiol 2011 64 401 406 21208779 10.1016/j.jclinepi.2010.07.015

[B24] Guyatt GH Oxman AD Vist GE Kunz R Falck-Ytter Y Alonso-Coello P Schünemann HJ GRADE: an emerging consensus on rating quality of evidence and strength of recommendations BMJ 2008 336 924 926 18436948 10.1136/bmj.39489.470347.AD PMC2335261

[B25] Moher D Liberati A Tetzlaff J Altman DG Group P Preferred reporting items for systematic reviews and meta-analyses: the PRISMA statement BMJ 2009 339 2535 PMC3090117 21603045

