# Supplementary material for: Antiviral efficacy and safety of abacavir-containing combination antiretroviral therapy as first-line treatment of HIV-infected children and adolescents: a systematic review protocol
Source: Syst Rev. 2014 Aug 12;3:87. doi: 10.1186/2046-4053-3-87 (PMC4137106; doi:10.1186/2046-4053-3-87)
Supplement: Additional file 1: — Medline search strategy using PubMed. [file 2046-4053-3-87-S1.doc]

**Additional file 1**

Medline search strategy using PubMed

#1 (HIV Infections[MeSH] OR HIV[MeSH] OR hiv[tw] OR hiv-1*[tw] OR hiv-2*[tw] OR hiv1[tw] OR hiv2[ tw] OR hiv infect*[tw] OR human immunodeficiency virus[tw] OR human immunedeficiency virus[tw] OR human immuno-deficiency virus[tw] OR human immune-deficiency virus[tw] OR ((human immun*) AND (deficiency virus[tw])) OR acquired immunodeficiency syndrome[tw] OR acquired immunedeficiency syndrome[tw] OR acquired immuno-deficiency syndrome[tw] OR acquired immune-deficiency syndrome[tw] OR ((acquired immun*) AND (deficiency syndrome[tw])) OR “sexually transmitted diseases, viral”[MESH:NoExp])

#2 (“Antiretroviral Therapy, Highly Active”[MeSH] OR “Anti-Retroviral Agents”[MeSH] OR “Antiviral Agents”[MeSH: NoExp] OR ((anti) AND (hiv[tw])) OR antiretroviral*[tw] OR ((anti) AND (retroviral*[tw])) OR HAART[tw] OR ((anti) AND (acquired immunodeficiency[tw])) OR ((anti) AND (acquired immunedeficiency[tw])) OR ((anti) AND (acquired immuno-deficiency[tw])) OR ((anti) AND (acquired immune-deficiency[tw])) OR ((anti) AND (acquired immun*) AND (deficiency[ tw])))

#3 ("child"[MeSH Terms] OR "child"[All Fields]) OR ("infant"[MeSH Terms] OR "infant"[All Fields]) OR

("adolescent"[MeSH Terms] OR "adolescent"[All Fields])

#4 ("pediatrics"[MeSH Terms] OR "pediatrics"[All Fields] OR "pediatric"[All Fields]) OR ("pediatrics"[MeSH Terms] OR "pediatrics"[All Fields] OR "paediatric"[All Fields])

#5 (#3 OR #4)

#6 abacavir OR ziagen OR 1592U89

#7 Search #1 AND #2 AND #5 AND #6
